# Effect of EMG-biofeedback robotic-assisted body weight supported treadmill training on walking ability and cardiopulmonary function on people with subacute spinal cord injuries – a randomized controlled trial

**DOI:** 10.1186/s12883-019-1361-z

**Published:** 2019-06-24

**Authors:** Eddy Yu Yeung Cheung, Kevin Ka Ki Yu, Rachel Lai Chu Kwan, Carmen Ka Man Ng, Rosanna Mei Wa Chau, Gladys Lai Ying Cheing

**Affiliations:** 1 Department of Rehabilitation Sciences, The Hong Kong Polytechnic University, Hong Kong Special Administrative Region, China; 2 Physiotherapy Department, Kowloon Hospital, Hospital Authority, Hong Kong Special Administrative Region, China; 3 University Research Facility in Behavioral and Systems Neuroscience, The Hong Kong Polytechnic University, Hong Kong Special Administrative Region, China; 4 Jockey Club School of Public Health and Primary Care, The Chinese University of Hong Kong, Hong Kong Special Administrative Region, China

**Keywords:** Locomotion, Independence, Oxygen consumption

## Abstract

**Background:**

Body weight supported treadmill training (BWSTT) is a frequently used approach for restoring the ability to walk after spinal cord injury (SCI). However, the duration of BWSTT is usually limited by fatigue of the therapists and patients. Robotic-assisted body weight supported treadmill training (RABWSTT) was developed to tackle the aforesaid limitation. Currently, limited randomized controlled trials are available to investigate its effectiveness, especially on cardiopulmonary function. The aim of this two-arm, parallel-group randomized controlled trial is to examine the feasibility of adapting an EMG-biofeedback system for assist-as-needed RABWSTT and its effects on walking and cardiopulmonary function in people with SCI.

**Methods:**

Sixteen incomplete SCI subjects were recruited and randomly allocated into an intervention group or control group. The intervention group received 30 min of RABWSTT with EMG biofeedback system over the vastus lateralis muscle to enhance active participation. Dose equivalent passive lower limbs mobilization exercise was provided to subjects in the control group.

**Results:**

Significant time-group interaction was found in the Walking Index for Spinal Cord Injury version II (WISCI II) (*p* = 0.020), Spinal Cord Independence Measure version III (SCIM III) mobility sub-score (*p* < 0.001), bilateral symmetry (*p* = 0.048), maximal oxygen consumption (*p* = 0.014) and peak expiratory flow rate (*p* = 0.048). Wilcoxon signed-rank test showed that the intervention group had significant improvement in the above-mentioned outcomes after the intervention except WISCI II, which also yielded marginal significance level.

**Conclusion:**

The present study demonstrated that the use of EMG-biofeedback RABWSTT enhanced the walking performance for SCI subjects and improve cardiopulmonary function. Positive outcomes reflect that RABSTT training may be able to enhance their physical fitness.

**Trial registration:**

The study protocol was approved by the Research Ethics Committee (Kowloon Central/ Kowloon East), Hospital Authority on 6 December 2013, and the Human Subjects Ethics Sub-committee of The Hong Kong Polytechnic University on 15 May 2013, with reference numbers KC/KC-13-0181/ER-2 and HSEARS20130510002 respectively. The study was registered in ClinicalTrials.gov on 20 November 2013, with reference number NCT01989806.)

## Background

Robotic-assisted body weight supported treadmill training (RABWSTT) has received much attention in gait rehabilitation for people with neurological conditions. Robotic orthoses provide guidance in the lower limbs movement during walking training that enables prolonged walking training with afferent input of normal gait pattern. This extensive exposure of task-specific repetitive training helps promote reorganization of the primary motor cortex [1], and functional outcomes can be improved in patients with neurological conditions like spinal cord injuries (SCI).

Randomized controlled trials [2, 3] have been performed to explore the effectiveness of RABWSTT in acute SCI subjects (less than 6 months post injury). Eight weeks of RABWSTT can result in improvement in walking independence, walking speed and lower limb muscle strength. However, in a randomized controlled trail (RCT) conducted by Niu [4] which involved chronic SCI subjects (more than 24 months post injury), no significant findings in walking performance was identified, although better improvement was found in subjects with higher walking capacity at the time of recruitment. It is possible that chronicity of injury may lead to different rehabilitation effects and limited evidences are available for the effect of RABWSTT on sub-acute SCI subjects (duration of injury from 6 to 24 months).

Additionally, participation of subjects during training is an essential component in stimulating neuroplasticity. Previous RCTs included complete passive guidance of lower limb [5–8] or minimal guidance without real time monitoring of muscle contraction [2, 3]. Muscle activation over lower limbs may be reduced which underestimated the effects of RABWSTT.

On the other hand, the vicious cycle of limitation in mobility state and reduction in cardiopulmonary function is frequently observed in people with SCI. Prolonged immobilization as well as reduced mobility states can lead to poor cardiopulmonary function. This limits social integration and increases the risk of developing other comorbidities including heart disease and pneumonia. Although a recent study has reported that RABWSTT was equivalent to exercise with moderate intensity [9], there is a lacking of RCT that investigates the effect of RABWSTT on cardiopulmonary function in SCI patients.

The aim of the current RCT is to investigate the feasibility of adapting an EMG-biofeedback system for assist-as-needed RABWSTT in people with SCI and its effects on walking and cardiopulmonary functions.

## Methods

### Subjects

Incomplete SCI subjects with age 18 or above were recruited into the study if they fulfilled the inclusion criteria (Table 1). There was no maximum age limit for the subjects. Subjects were excluded from the study if they did not satisfy the criteria of RABWSTT system (Table 1). Written consent was signed by eligible participants prior to the entry of study.

**Table 1:** Inclusion and Exclusion Criteria

Inclusion Criteria	Exclusion Criteria
• Suffering from incomplete spinal cord injury with classification B, C or D under the International Standards for Neurological Classification of Spinal Cord Injury (ISNSCI) • Lesion level at or above L5 • 6–24 months post injury • Non-progressive lesion • Able to tolerate tilt-table standing in 90 degrees for more than 30 min • Able to walk disregarding the use of aids / assistance.	• Contraindications of Lokomat system^a^, including • Orthosis cannot be adjusted to fit the body (lower limbs) • Body weight greater than 135 kg • Severely fixed contractures • Bone instability (non-consolidated fractures, unstable spinal column, severe osteoporosis) • Open skin lesions in the area of the lower limbs and torso • Circulatory problems • Cardiac contraindications • Uncooperative or self-harming behavior, such as transitory psychotic syndrome • Severe cognitive deficits • Patients with (long-term) infusions • Mechanical ventilation • Patients with extremely disproportionate growth of the legs or spinal column • Severe vascular disorders of the lower limbs • Patients who have been ordered to remain in bed or immobile • Hip, knee, ankle arthrodesis

^**a**^Adopted from Lokomat instructions for use, 2011 [10]

Demographic data, including sex, age, duration and severity of injury, was collected and subjects were randomly allocated into an intervention group or control group by using sealed envelopes. One of them indicated intervention group while the other one indicated control group. The allocation procedure was performed by a research assistant blinded to data collection and training procedures. Subjects were blinded to group allocation. Outcome measures were collected within 1 week before the start of intervention and reassessment was performed within 1 week after the subjects completed their 8 weeks of intervention by an independent assessor who was blinded for the group allocation.

### Treatment protocols

One hour of standard physiotherapy program, including limbs mobilization and strengthening, trunk stabilization, wheelchair maneuver training and overground walking training was provided to all subjects twice per week, 60 min per session. Thirty minutes (exclude set-up time) of RABWSTT or passive lower limb mobilization 3 times per week for 8 weeks was given to subjects on top of the standard physiotherapy program based on their group allocation. Subjects in both groups were instructed by the same trainer.

Lokomat system^1^ was used for RABWSTT. Body weight support was set at 40% of body weight to minimize individual difference. Training speed was adjusted to comfortable speed. Assist-as-needed guidance force was given to aid proper walking pattern. EMG-biofeedback system^2^ was applied to the bilateral vastus lateralis of subjects. We selected the vastus lateralis muscle because this one-joint muscle is the largest part of the quadriceps muscle responsible for standing leg stance support during walking. Audio feedback was generated if the muscle activation was less than 30% of maximal recruitment to encourage active participation during the stance phase of the gait cycle. A physiotherapist monitored the participation of subjects in the first 2 sessions to make sure they could correctly follow the biofeedback.

Passive lower limbs mobilization training^3^ by using lower limb active-passive exerciser was given to the control group. We chose this as the control because we would like to provide robot-guided lower extremities movement with minimal locomotion-related afferent input, which can minimize the difference in improvement in outcomes due to the repetitive lower limbs guided movements. They were asked to sit on a high chair and to relax. The exerciser performed passive lower limb cycling exercise for the subjects.

### Outcome measures

Primary outcomes including Walking Index for Spinal Cord Injury version II (WISCI II) and Spinal Cord Independence Measure version III (SCIM III), which assess walking independence [11] and functional independence [12] respectively, were used.

Lower limb muscle strength was tested by manual muscle testing based on the Medical Research Council scale for muscle strength. The scores from the 10 key muscles were added up to form the lower extremity motor score (LEMS) [13]. We intentionally used LEMS as subjects experience muscle weakness at different muscle groups and at different sides. Lower limb-force (L-force) function in Lokomat system was also used. Isometric muscle strength of hips and knees were measured by force sensors inside orthoses of the system. Muscle spasticity was assessed by the Modified Ashworth Scale. Hip and knee flexors and extensors were tested. Joint stiffness was measured by the Lower limb-stiff (L-stiff) function of Lokomat system. Passive hip and knee joint movements in different speeds were provided and resistive torque was assessed to reflect joint stiffness.

Quality of gait pattern was assessed by gait analysis system.^4^ Subjects walked on a pressure-detectable walkway at their comfortable speed which allowed the use of assistance and orthoses. Subjects were asked to start walking two meters away from the walkway for acceleration and to walk two more meters after walking through the walkway for deceleration. Walking speed, heel-heel base support, bilateral stance duration and bilateral symmetry (ratio of stride length of two legs) were captured for further data analysis.

Submaximal exercise stress test with the use of upper limb ergometer exercise was used to estimate maximal oxygen consumption. Subjects were asked to perform graded upper limb ergometer exercise with a gas analysis system.^5^ Oxygen uptake during the test was analyzed by the system and maximal oxygen consumption is estimated based on body weight, age and heart rate of subject. The following protocols were adopted for the gas analysis test: one minute of rest followed by with one minute of warm-up using the least resistance, and increased the resistance by 1 level per one-minute time. The test was stopped when either the heart rate reached 85% of maximal heart rate (maximal heart rate was determined by 220-age) [14], or the subject could not tolerate the test.

Spirometry^6^ was used to assess the respiratory function of subjects. Peak expiratory flow (PEF), forced expiratory volume in first second (FEV_1_) and forced vital capacity (FVC) were assessed. One-minute rest interval was provided between trials to prevent hyperventilation.

### Data analyses

Statistical analyses were performed with the Statistical Package for the Social Science software. Baseline comparisons were assessed by Chi-Square test and Mann Whitney-U test. Two-way repeated measures ANOVA was used to analysis the change in each outcome measure among two groups and time. Subsequent analyses were conduction separately for the groups and time when an overall significant effect was detected by ANOVA in the timeXgroup interaction test. Wilcoxon signed-rank test with Bonferroni correction for pairwise comparison instead of ANOVA was used for MAS as it is an ordinal outcome. Intention-to-treat analysis using the last observation carried forward (LOCF) method was adopted for missing or dropped out data.

Correlation of WISCI II with the LEMS, L-force and parameters of quality of gait was also assessed by Pearson’s correlation coefficient in order to assess the association between walking independence with lower limb muscle strength and gait related parameters. Data for both pre-intervention and post-intervention were used. An alpha value of 0.05 was set for all the tests.

### Sample size calculation

A pilot study involving 6 subjects, 3 in each group, was performed before the study. Among the three-main functional related outcomes (WISCI II, SCIM III and maximal oxygen consumption), maximal oxygen consumption yielded the smallest partial eta-square of 0.49. Sample size was calculated by using G*Power 3.0 software (Power = 0.8, alpha = 0.05). With an addition of 10% of subject recruitment for possible dropout, a total of 16 subjects with 8 subjects in each group was calculated.

## Results

**Fig. 1:**
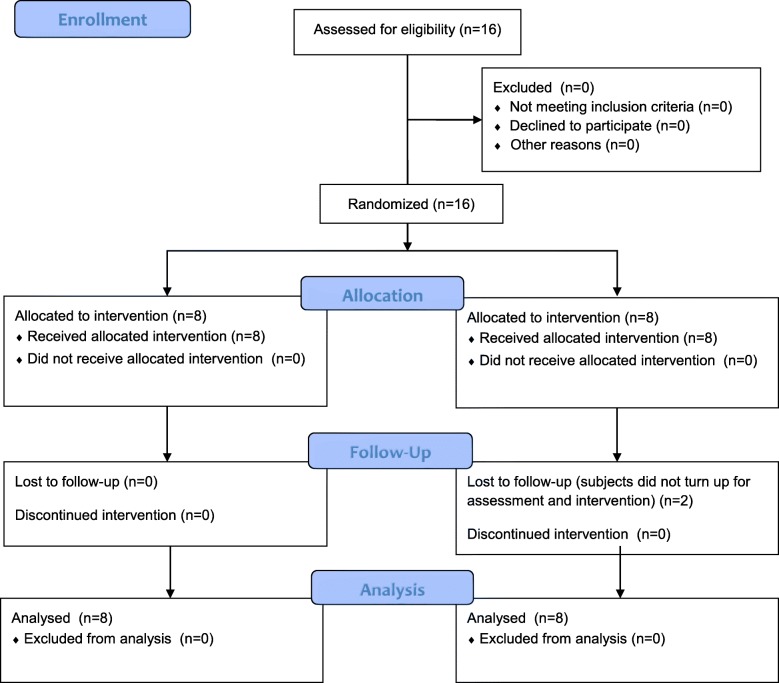
Flow of the study

Sixteen incomplete SCI subjects were recruited from a tertiary hospital in Hong Kong from April 2014 to July 2015. The mean age was 54.3 ± 9.6 years with mean duration of injury 13.7 ± 7.4 months. Two subjects in the control group failed to follow-up. (The flow of the study can be referred to Fig. 1.) The demographics of the two groups of subjects are shown in Table 2. The mean age of the subjects was 54.3 years old.

**Table 2:** Demographic data of subjects

Demographic data	RABWSTT group(*n* = 8)	Control group(n = 8)	*p*-value
Age (years)	55.6 ± 4.98	53.0 ± 12.94	0.875
Sex (male)	87.5%	50%	0.134
BMI (kg/m^2^)	23.4 ± 2.50	22.0 ± 3.75	0.529
Level of injury	C1-L2	C3-L2	0.671
ISNSCI classification	C: 7	C: 4	0.134
	D: 1	D: 4	
Duration of injury (months)	17.0 ± 7.01	10.4 ± 6.31	0.130
	Range: 6–23	Range: 6–23	

*BMI* Body mass index, *ISNSCI* International standards for neurological classification of spinal cord injury, *RABSWTT* Robotic-assisted body weight supported treadmill training

No adverse effect or discomfort was reported by subjects. Tables 3, 4, 5 and 6 shows the evaluation results of outcome measures. Significant timeXgroup interaction was found in WICSI II (*p* = 0.02), mobility sub-score of SCIM III (*p* < 0.001), bilateral symmetry(*p* = 0.048), maximal oxygen consumption (*p* = 0.014) and PEF (*p* = 0.048). Wilcoxon signed-rank test with Bonferroni correction showed significant improvements in RABWSTT group in the abovementioned outcomes (*p* < 0.025) except for WISCI II which also reached marginal significance (*p* = 0.027), but none of these outcome measures were found to be improved in control group. No significant timeXgroup interaction was found in other outcomes with no significant between group difference(*p* > 0.05). On the other hand, however, there was no significant timeXgroup interaction or between group difference detected after the intervention period for L-stiff and Modified Ashworth Scale over lower limbs muscles (*p* > 0.05).

**Table 3:** Evaluation of walking independence, functional independence and lower limbs muscles strength

	RABWSTT group	Control group
WISCI II		
Baseline	14.6 ± 4.27	17.0 ± 2.78
Posttreatment	16.3 ± 4.95	17.1 ± 2.59
Overall within-group effect: *p* = 0.003^*^; overall between-group effect: *p* = 0.382; time X group interaction: *p* = 0.020^*^		
SCIM III self-care		
Baseline	15.3 ± 6.59	16.8 ± 4.98
Posttreatment	15.1 ± 5.79	16.8 ± 4.98
Overall within-group effect: *p* = 0.854; overall between-group effect: *p* = 0.585; time X group interaction: *p* = 0.854		
SCIM III respiratory and sphincter		
Baseline	34.0 ± 6.78	34.9 ± 7.68
Posttreatment	32.6 ± 8.09	34.9 ± 7.68
Overall within-group effect: *p* = 0.623; overall between-group effect: *p* = 0.665; time X group interaction: *p* = 0.623		
SCIM III mobility		
Baseline	24.0 ± 12.13	28.4 ± 7.52
Posttreatment	28.6 ± 13.02	28.6 ± 7.80
Overall within-group effect: *p* = < 0.001^*^; overall between-group effect: *p* = 0.680; time X group interaction: *p* = < 0.001^*^		
SCIM III total		
Baseline	73.3 ± 19.73	80.0 ± 17.44
Posttreatment	71.0 ± 26.32	80.3 ± 17.69
Overall within-group effect: *p* = 0.817; overall between-group effect: *p* = 0.409; time X group interaction: *p* = 0.772		
LEMS		
Baseline	35.5 ± 4.50	39.4 ± 9.07
Posttreatment	36.5 ± 6.16	40.0 ± 8.49
Overall within-group effect: *p* = 0.043^*^; overall between-group effect: *p* = 0.326; time X group interaction: *p* = 0.616		
L-force		
Baseline	302.4 ± 126.00	227.9 ± 59.15
Posttreatment	341.0 ± 111.02	228.4 ± 65.61
Overall within-group effect: *p* = 0.112; overall between-group effect: *p* = 0.061; time X group interaction: *p* = 0.121		

*LEMS* Lower extremity motor score, *RABWSTT* Robotic-assisted body weight supported treadmill training, *SCIM III* Spinal Cord Independence Measure Version III, *WISCI II* Walking Index for Spinal Cord Injury version II; ^*^: *p*-value< 0.05

L-force was measured over 4 muscle groups in each leg: Hip flexors, hip extensors, knee flexors and knee extensors. The values from left leg and right leg were added up for analysis

**Table 4:** Evaluation of gait parameters

	RABWSTT group	Control group
Walking speed (cm/s)		
Baseline	43.8 ± 24.31	43.9 ± 28.97
Posttreatment	44.7 ± 23.97	48.1 ± 34.10
Overall within-group effect: *p* = 0.261; overall between-group effect: *p* = 0.901; time X group interaction: *p* = 0.445		
Heel-heel base support (cm)		
Baseline	9.9 ± 4.77	14.1 ± 5.05
Posttreatment	10.2 ± 4.90	13.5 ± 5.78
Overall within-group effect: *p* = 0.887; overall between-group effect: *p* = 0.154; time X group interaction: *p* = 0.575		
Bilateral stance duration (%)		
Baseline	50.6 ± 22.90	51.9 ± 15.33
Posttreatment	50.7 ± 21.98	51.7 ± 16.55
Overall within-group effect: *p* = 0.921; overall between-group effect: *p* = 0.908; time X group interaction: *p* = 0.767		
Bilateral symmetry (%)		
Baseline	0.8 ± 0.20	1.0 ± 0.03
Posttreatment	0.9 ± 0.20	1.0 ± 0.03
Overall within-group effect: *p* = 0.017^*^; overall between-group effect: *p* = 0.138; time X group interaction: *p* = 0.048^*^		

RABWSTT: Robotic-assisted body weight supported treadmill training; ^*^: *p*-value< 0.05

**Table 5:** Evaluation of maximal oxygen consumption and pulmonary functions

	RABWSTT group	Control group
Maximal oxygen consumption (L/kg/min)		
Baseline	25.7 ± 7.16	20.5 ± 2.93
Posttreatment	26.4 ± 6.99	20.5 ± 2.84
Overall within-group effect: *p* = 0.012^*^; overall between-group effect: *p* = 0.057; time X group interaction: *p* = 0.014^*^		
Peak expiratory flow (L)		
Baseline	5.0 ± 2.34	4.7 ± 2.34
Posttreatment	5.7 ± 2.25	4.7 ± 2.58
Overall within-group effect: *p* = 0.077; overall between-group effect: *p* = 0.584; time X group interaction: *p* = 0.048^*^		
Forced expiratory volume in first second (FEV_1_)		
Baseline	2.2 ± 0.81	2.2 ± 0.81
Posttreatment	2.4 ± 0.94	2.2 ± 0.91
Overall within-group effect: *p* = 0.204; overall between-group effect: *p* = 0.792; time X group interaction: *p* = 0.104		
Forced vital capacity (L)		
Baseline	2.7 ± 1.30	2.6 ± 0.75
Posttreatment	2.7 ± 1.26	2.6 ± 0.93
Overall within-group effect: *p* = 0.360; overall between-group effect: *p* = 0.849; time X group interaction: *p* = 0.872		

RABWSTT: Robotic-assisted body weight supported treadmill training; ^*^: *p*-value< 0.05

**Table 6:** Post-hoc analysis for outcomes with significant timeXgroup interaction (Wilcoxon signed-rank test with Bonferroni correction, *α* =0.025)

	RABWSTT group	Control group
WISCI II		
Baseline	14.6 ± 4.27	17.0 ± 2.78
Posttreatment	16.3 ± 4.95	17.1 ± 2.59
Within-subject *p*	0.027	0317
SCIM III mobility		
Baseline	24.0 ± 12.13	28.4 ± 7.52
Posttreatment	28.6 ± 13.02	28.6 ± 7.80
Within-subject *p*	0.011^*^	0.317
Bilateral symmetry (%)		
Baseline	0.8 ± 0.20	1.0 ± 0.03
Posttreatment	0.9 ± 0.20	1.0 ± 0.03
Within-subject *p*	0.018^*^	0.345
Maximal oxygen consumption (L/kg/min)		
Baseline	25.7 ± 7.16	20.5 ± 2.93
Posttreatment	26.4 ± 6.99	20.5 ± 2.84
Within-subject *p*	0.018^*^	0.916
Peak expiratory flow (L)		
Baseline	5.0 ± 2.34	4.7 ± 2.34
Posttreatment	5.7 ± 2.25	4.7 ± 2.58
Within-subject *p*	0.017^*^	0.674

*RABWSTT* Robotic-assisted body weight supported treadmill training; ^*^: *p*-value< 0.025

Table 7 shows the result of correlation between WISCI II and lower limb muscles strength and gait-related parameters. It is found that LEMS(r = 0.610, *p* < 0.001), walking speed(r = 0.715, *p* < 0.001), bilateral stance duration(r = − 0.761, *p* < 0.001) and bilateral symmetry(r = 0.460, *p* = 0.008) were correlated with WISCI II with fair to moderately strong correlation.

**Table 7:** Pearson’s correlation coefficient between WISCI II with lower limb muscles strength and gait-related parameters

	Pearson’s r	*p*-value
LEMS	0.610	< 0.001^*^
L-force	−0.045	0.805
Walking speed (cm/s)	0.715	< 0.001^*^
Heel-heel base of support (cm)	− 0.217	0.234
Bilateral stance duration (%)	−0.761	< 0.001^*^
Bilateral symmetry (%)	0.460	0.008^*^

*LEMS* Lower extremity motor score; L-force: Lower limb-force; ^*^: *p*-value < 0.05

### Discussion

The primary goal of RABWSTT is to improve the walking ability for patients, and our results supported its use in SCI subjects. The mean change in WISCI II were 6–12 in acute subjects [2, 3] and 1 in chronic subjects [15]. The marked improvement in acute subjects may be partly related to natural recovery of nervous system (the control counterparts yielded 5–6 improvement in WISCI II), and partly because of the improvement in lower limb muscle strength. In the current study, we yielded an average improvement of 1.5 in WISCI II, which is similar to those in chronic subject groups. However, in these studies, there is significant improvement in the lower limb muscle strength, which is an important determining factor of independent walking [16]. In addition, we also detected an improvement in walking independence in terms of mobility sub-score of SCIM III despite no significant improvement in muscle strength. This suggests that RABSWTT can help promote walking independence in subacute SCI subjects by means of improvement other than muscle strength. This finding evidently shows that RABWSTT is effective in enhancing mobility independence, which is independent to the duration of injury.

The same applies to the motor training in most neurological diseases - high repetition of task-specific training with proper sensory feedback are essential elements for neuroplasticity after SCI [17]. An EMG-biofeedback system was implemented in our training protocol to enhance muscle contraction. It has been proven that visual and audio-feedback can promote muscle recruitment [18], increase muscle performance [18], and promote better improvement in lower limbs performance in people with neurological conditions [19]. All of our subjects could follow the system to participate in the walking training within the first two sessions of training and thus met sufficient amounts of active muscle recruitment.

Some authors proposed that trainings with suitable difficulty, variables and allowance of errors are necessary in motor learning in walking function [20–22]. This may explain why in Field-Fote’s study group [6, 8], they yielded better outcomes in the overground walking groups than RABWSTT group as they implemented full guidance during RABWSTT which minimizes participation and variability. In contrast, we implemented the use of EMG-biofeedback system over lower limb muscles to monitor as well as encourage muscle contraction to enhance modulation in central nervous system without any adverse effect, indicating that it is a feasible and safe way to promote participation of subjects during training. In addition, we only provided assist-as-needed guidance from robotic orthoses, for which variance in stepping was ensured during training.

Although RABWSTT can elicit neuroplasticity [23] to promote walking ability, it is still uncleare by what means the improvement can be achieved. Varoqui [24] has proposed that the change in ankle properties is one of the reasons of normalization of gait pattern, yet the effect of RABWSTT on ankle properties is questionable as only passive dorsiflexion was given during the training without repetitive afferent input of normal walking pattern. In our study, we found that both LEMS, walking speed, bilateral stance duration and bilateral symmetry were associated with walking independence but only bilateral symmetry was improved after training, which was achieved mainly by the normalization of hip and knee joints movement during the swing phase. We proposed that the improvement of walking independence after RABWSTT may come from the improvement of bilateral symmetry. Repetitive movement of both lower limbs with similar stride length was guaranteed by the robotic system via guidance to hip and knee joints. Proprioceptive senses from these two joints helped learn a normal gait pattern and in turn improved walking independence, which matches the notion of neuroplasticity. However, further study investigate of a possible causal relationship between walking independence and gait parameters is needed to prove the hypothesis.

In contrary to RABWSTT group, we noted that the control group showed marginal significant improvement in LEMS after the intervention period (*p* = 0.059). The improvement may be partly because of the muscle strengthening component in the conventional training, but partly also because of the relatively shorter duration of injury. It is well known that functional recovery is faster early post-injury and gradually slows down [25]. The duration of injury was 7 months shorter in the control group, a marginal significant difference (*p* = 0.130), which may account for the better improvement in LEMS in response to the conventional training.

Another focus of our study is to investigate the effect of RABWSTT on cardiopulmonary function. The results also supported that RABWSTT is effective in improving cardiovascular and pulmonary function in SCI subjects. Kressler [7] did not find a significant effect on peak oxygen consumption after RABWSTT. One of the possible reasons is the passive setting of RABWSTT in their study. Our study adapted assist-as-needed training with EMG-biofeedback system which can minimize passive guidance from robotic systems which reduces metabolic cost [26]. This can promote consumption of oxygen at the cellular level over peripheral muscles to enhance oxygen drive and to ensure the training provides sufficient stress to promote cardiopulmonary fitness. Moreover, the improved respiratory muscle strength, as reflected by PEF, helps deepen and quicken breathing rate during exercise leading to increased oxygen uptake [27].

The testing procedure may also be another reason for variations in results found. A recently published study showed significant improvement in peak oxygen consumption when subjects were tested in RABWSTT system but not in arm ergometer exercise [28]. Maximal oxygen consumption is not only determined by respiratory and cardiac muscles strength, but also by the utilization of oxygen in mitochondria. The subjects might have learned a way to cooperate with the robotic system to contract lower limbs muscles during guidance from orthoses after weeks of training. The improvement in peak oxygen consumption may come from the increase in muscle recruitment during walking exercise instead of improvement of cardiopulmonary system. However, we found that peak oxygen consumption was promoted in RABWSTT group during upper limb ergometer exercise, in which there was no extra upper limb strengthening component as compared to control group. The improvement is thus not likely related to increment in upper limb muscle recruitment, but comes from better capacity to utilize oxygen during intensive exercise training. Our present findings provide the first evidence to prove that RABWSTT not only shows task-specific improvement in exercise tolerance, but also provides a general training effect on cardiopulmonary system.

As locomotion of organism is to transfer oneself from one place to another, walking endurance should be one of the most important functional outcomes to reflect improvement in locomotor ability as well as quality of life. We have succeeded in showing that RABWSTT is effective in promoting walking independence and exercise tolerance, yet we have not implemented any assessment of measuring walking endurance, although several studies [2, 3] supported the use of RABWSTT in promoting walking endurance. Further studies are warranted to study the effects of RABWSTT on walking endurance and quality of life in subacute and chronic SCI subjects. On the other hand, mobility is a complex matter as changes in movement pattern is essential to perform daily tasks. However, the robotic system we used cannot provide varying environments during training. Future studies investigating the effect on the use of robotic training with varying environmental factors are suggested to bridge RABWSTT from task-specific training to task-oriented training.

Despite the small sample size, we have found significant improvement in walking independence, better gait control and increased cardiopulmonary function. Although we tried to implement intention-to-treat analysis to minimize the effects of dropout, significant dropout in the control group lowers the effect of intention-to-treat analysis that leads to possible bias in results. Discrepancies among patients such as functional ability before injury and at recruitment, injury mechanisms and surgical procedures may also affect the prognosis that limits the generalizability of our current results. Further studies with larger sample size are warranted to support the current result and to investigate the relationship between gait pattern and the improvement in walking ability after RABWSTT.

## Conclusions

The use of EMG-biofeedback for monitoring of subject participation during RABWSTT is a feasible treatment regime for promoting independent walking ability, equalizing bilateral limbs step length as well as enhancing maximal oxygen consumption and strengthening pulmonary muscles in people with incomplete SCI. This can help improve independence in daily activities in people with SCI, and enables them to enhance their walking endurance that promotes social re-integration.

## Data Availability

The datasets used and/or analyzed during the current study are available from the corresponding author on reasonable request.

## Abbreviations

BWSTT: Body weight supported treadmill training
FEV_1_: Forced expiratory volume in first second
FVC: Forced vital capacity
LEMS: Lower extremity motor score
L-force: Lower limb-force
L-stiff: Lower limb-stiff
PEF: Peak expiratory flow
RABWSTT: Robotic-assisted body weight supported treadmill training
RCT: Randomized controlled trial
SCI: Spinal cord injury
SCIM III: Spinal Cord Independence Measure version III
WISCI II: Walking Index for Spinal Cord Injury version II

## Footnotes

1 RABWSTT system: Lokomat V6, Hocoma AG, Switzerland

2 EMG-biofeedback system: Pathway MR-20 dual channel surface EMG system, The Prometheus Group, The United States

3 Lower limb active-passive exerciser: Motomed Vivo 2, RECK, Germany

4 Gait analysis system: GAITRite System, CIR System, Inc., US

5 Gas analysis system: Fitmate pro, COSMED, Italy

6 Spirometer: Pony FX, COSMED, Italy
